# Potentials and future perspectives of multi-target drugs in cancer treatment: the next generation anti-cancer agents

**DOI:** 10.1186/s12964-024-01607-9

**Published:** 2024-04-15

**Authors:** Ali Doostmohammadi, Hossein Jooya, Kimia Ghorbanian, Sargol Gohari, Mehdi Dadashpour

**Affiliations:** 1 Nervous System Stem Cells Research Center, Semnan University of Medical Sciences, Semnan, Iran; 2 Student Research Committee, Semnan University of Medical Sciences, Semnan, Iran; 3 Biochemistry Group, Department of Chemistry, Faculty of Science, Ferdowsi University of Mashhad, Mashhad, Iran; 4 Department of Biology, Central Tehran Branch, Islamic Azad University, Tehran, Iran; 5 Department of Medical Biotechnology, Faculty of Medicine, Semnan University of Medical Sciences, Semnan, Iran; 6 Cancer Research Center, Semnan University of Medical Sciences, Semnan, Iran

**Keywords:** Cancer treatment, Drug resistance, Polypharmacology, Multi-target drugs

## Abstract

Cancer is a major public health problem worldwide with more than an estimated 19.3 million new cases in 2020. The occurrence rises dramatically with age, and the overall risk accumulation is combined with the tendency for cellular repair mechanisms to be less effective in older individuals. Conventional cancer treatments, such as radiotherapy, surgery, and chemotherapy, have been used for decades to combat cancer. However, the emergence of novel fields of cancer research has led to the exploration of innovative treatment approaches focused on immunotherapy, epigenetic therapy, targeted therapy, multi-omics, and also multi-target therapy. The hypothesis was based on that drugs designed to act against individual targets cannot usually battle multigenic diseases like cancer. Multi-target therapies, either in combination or sequential order, have been recommended to combat acquired and intrinsic resistance to anti-cancer treatments. Several studies focused on multi-targeting treatments due to their advantages include; overcoming clonal heterogeneity, lower risk of multi-drug resistance (MDR), decreased drug toxicity, and thereby lower side effects. In this study, we'll discuss about multi-target drugs, their benefits in improving cancer treatments, and recent advances in the field of multi-targeted drugs. Also, we will study the research that performed clinical trials using multi-target therapeutic agents for cancer treatment.

## Introduction

Cancer is a leading cause of worldwide death and the most prevalent disease, with an estimated 19.3 million new cancer cases around the world in 2020 [1]. Therefore, its early detection and effective treatment development are crucial for managing this life-threatening disease.

Limitations of conventional chemotherapeutic agents, lack of specificity in existing epigenetic targeting drugs, and drug resistance are among the main challenges in cancer therapy [2, 3]. Through decades, different strategies have been developed for cancer treatment such as immunotherapy, gene therapy, epigenetic therapies, etc. [4–6]. While numerous cancer types may initially respond to chemotherapy, they can eventually develop resistance to it [7]. The ability of cancer cells to develop resistance against traditional treatments, and the growing number of drug-resistant cancers highlights the need for more research and the development of new treatments [8].

Targeted therapy, also known as precision medicine, blocks cancer cell growth by interfering with specific molecules needed for cancer development and growth, instead of simply interfering with all rapidly dividing cells like traditional chemotherapy [9]. Tamoxifen was the first targeted cancer therapy approved in the 1970s. It blocks the growth of estrogen receptor (ER)-positive breast cancer cells by binding to the estrogen receptor and preventing estrogen from binding [10]. The targeting therapy can be classified into single- and multi-targeting agents.

Single-target therapy has been a major advance in cancer treatment, but it has limitations. When single-target therapy fails, the alternative strategy is multi-target therapy, which includes polypharmacological drugs or drug combinations [11].

Polypharmacology involves targeting multiple tumor growth and progression-related pathways, making it more effective in treating complex diseases and drug-resistant cancers. Studies in polypharmacology could reveal new off-targets for current drugs, offering insight into drug side effects and toxicities. Furthermore, it can aid drug repurposing by identifying new indications or therapeutic targets for existing drugs [12]. Despite the optimistic outlook on multi-target therapy, overcoming challenges such as appropriate target selection is crucial for enhancing treatment efficacy [13].

Herein, we'll dive into the concept of polypharmacology, its potential, challenges, and future perspectives. Also, we'll argue the recent multi-target drug studies and potential therapeutic targets for developing anticancer agents in few prevalence malignancies.

### Single-, combination-, and multi target directed ligands-therapies; what's the difference?

Cancer treatments can be categorized based on the way therapeutic agents are employed into single agents, combination, and multi-target directed ligands (MTDLs) which are described as follows.

Monotherapy, also known as single-target therapy, aims to combat cancer by selectively attacking certain genes and proteins responsible for the survival and proliferation of malignant cells [14]. Unlike conventional chemotherapy drugs that exhibit a lack of selectivity towards cancer cells versus normal cells, this method ensures reduced harm to healthy cells, consequently minimizing the occurrence of substantial toxicity and side effects [15]. While monotherapy has shown some efficacy in certain cases, it may not be effective for all patients because the tumor cells can become resistant to monotherapies [16].

Combination therapy is a therapeutic modality that employs combining two or more agents with different mechanisms of action to achieve synergistic effects against cancer [17]. Since the discovery of new pharmacological anti-cancer agents is arduous and costly, it is essential to identify more effective methods that are economically viable [18]. While monotherapy is still applicable in some cases, combination therapy is increasingly recognized for its effectiveness and broad treatment coverage in managing complex diseases like cancer [19]. However combination therapy is a feasible option, there are remain challenges such as cost-effectiveness [20], and identifying the best drug combinations [21] which will be discussed in the next section.

Drug resistance is an important issue with current treatments which can be overcome using MTDLs [22]. MTDLs are a new class of drugs that target multiple receptors/enzymes simultaneously leading to better efficacy, preventing drug resistance development, and also combating it [23]. This strategy also has the potential to lower the required dosage of individual drugs, reducing the risk of adverse effects and enhancing treatment outcomes [7]. On the other hand, designing selective MTDLs with high affinity to their targets while avoiding off-target effects is a significant challenge in MTDLs treatments [24]. Understanding the pharmacokinetics (PK) and pharmacodynamics (PD) of designed ligands is another challenge, however, computer-aided drug designing tools provided applications for describing PK (i.e. absorption, distribution, metabolism, excretion, and toxicity (ADMET) properties) of designed drugs more predictably [25]. MTDLs reveal great superiority in comparison to mono- & combination-therapies which will further be discussed.

### Cancer and the necessity of using multi-targeted drugs

Cancer resistance is affected by Darwinian law, intra-tumor cell heterogeneity, and compensatory pathways often result in the tumor cells' survival [26]. It is the main challenge with monotherapies [8], which is attributed to up to 90% of cancer-associated deaths [27] and can be caused by various factors. Under the treatment pressure, cancer cells can adapt molecular and cellular mechanisms to evade the effects of the drug, often evolve into more aggressive or metastasis phenotypes, and limit the success of monotherapies [28].

Combination therapies have shown great potential for cancer treatment reducing monotherapy's defects [29–31]. They improve treatment outcomes, lead to synergistic anticancer effects, overcome clonal heterogeneity, and reduce drug resistance probability [32–34]. However, it's challenging to identify an effective combination [21, 35]. Combination therapy can also lead to side effect accumulation. They may include the sum of each drug's known side effects or completely unexpected side effects caused by drug-drug interactions [17, 36]. The treatment-related adverse events resulting from combination therapy had led to dose reduction or discontinuation reported in several studies [37–41]. Therefore, computational methods are employed to predict the right combinations for cancer treatment [42, 43]. In comparison, the MTDLs are constructed of a single compound designed for multifunctional properties with fewer side effects and more predictable toxicity. Also, combination therapies indicated higher utility values than monotherapies but they were generally more expensive [38]. While the MTDLs can modulate multiple targets simultaneously making it cost-effective as monotherapies and high efficacy as combination therapies in administration for patients [44]. Additionally, the potential for useful drug combinations is restricted by the risk of side effects, drug interactions, and technological challenges in obtaining stable pharmaceuticals. However, in theory, the number of useful combinations is unlimited if the molecular structure is properly selected and optimized. Also, in practice, it is most feasible to obtain ligands based on two to five pharmacophores [45]. Moreover, the drugs regimen of a combination therapy can indicate different absorption and distribution profiles which can affect the treatment outcomes. Also, administering doses or timings for agents of a combination treatment regimen with different half-lives is also challenging [46]. In contrast, using the computational approaches in designing MTDLs provides more predictable PK & physicochemical features resulting in more desirable ADMET profile of designed drugs [47]. In addition, it's generally easier to optimize the dose for a multi-targeted ligand than to do so separately for the components of the combination therapy regimen. Lastly, the clinical trial approval in a combination therapy requires each drug to be investigated separately, and then in combination with each other which is cost- and time-consuming while, MTDLs are time- & cost-efficient for clinical trials since a single compound is involved in the study [45].

Overall, multi-targeted treatments, specifically MTDLs, can provide several benefits in cancer treatment leading to improved overall survival with decreased side effects for cancerous patients.

### Strategies for developing MTDLs

The methods commonly used to develop MTDLs can be classified into two categories. The first category involves a random screening approach, while the second category utilizes a knowledge-based approach to combine scaffolds from different active molecules with known activity against a particular target. This latter approach is referred to as the framework combination approach [48].

Random screening involves using quantitive structure–activity relationship (QSAR) and/or virtual screening to discover an anti-cancer agent. QSAR serves as a valuable tool for uncovering the interplay between structure and activity within smaller congeneric compound series and enables the understanding of physicochemical and biological properties of the molecules for further targeting in cancer treatment [49, 50]. On the other hand, virtual screening allows for the docking of thousands or even millions of compounds to bind to proteins associated with cancer in a relatively cost-effective way. By doing so, it can help in the discovery of potential inhibitors for specific proteins or entire signaling pathways involved in the development of cancer [51].

The framework combination approach is a knowledge-based method to discover multi-target drugs by combining drugs/pharmacophores for developing a new hybrid molecule with the desired activity toward multiple targets [52]. The molecular components or individual partners can come together covalently to form a molecular matrix, by fusing, merging, or linking [53].

**Fig. 1:**
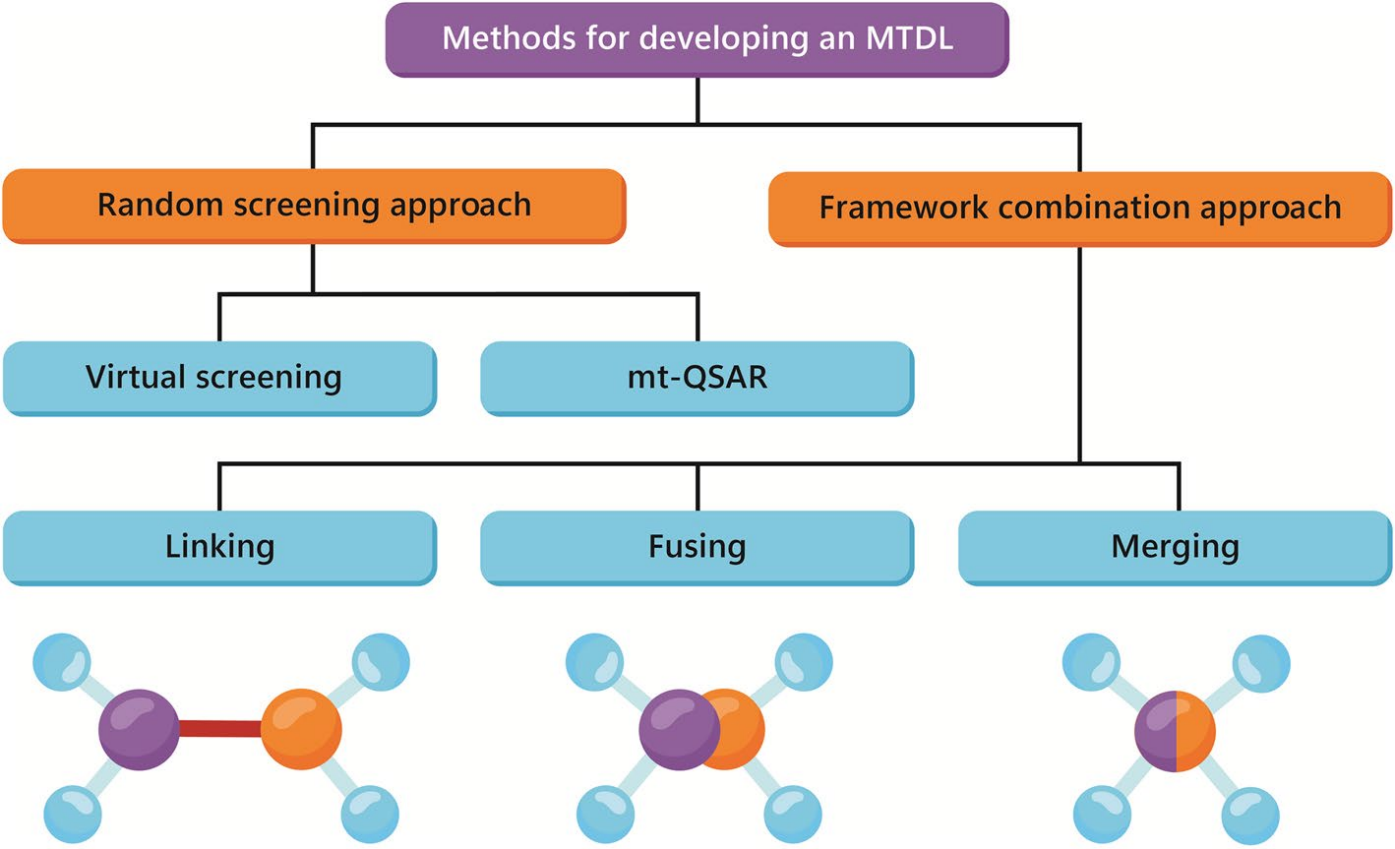
The schematic view of strategies that have been utilized to develop multi-target anti-cancer agent to combat cancer

The fused strategy combines two or more distinct biologically active pharmacophoric moieties, usually via a zero-length linker or a spacer, to form a new molecular hybrid [54]. While merged strategy involves merging pharmacophores into one molecule, resulting in the development of a unique, smaller chemical compound with retained pharmacological properties but notably different chemical traits [55]. The combined agents might hold onto the functional properties of one or both of the overlapping drugs [54]. Also, this strategy can lead to a resulting compound with reduced molecular weight compared to fusing/linking strategies employment [56]. Furthermore, the merging requires in-depth knowledge about the side chains interaction and the conformation that affects the compound function while the linking strategy is simpler [57]. The linking is the binding of two compounds that bind within their pharmacophores together through a linker (cleavable or not) to obtain a new compound capable of aiming multiple targets at the same time [58, 59]. For example, trastuzumab emtansine, an FDA-approved drug [60], is an MTDL that linked an anti-HER2/neu antibody with emtansine (a microtubule inhibitor) through MCC (4-[N-maleimidomethyl]cyclohexane-1-carboxylate) linker [45]. Furthermore, the linking strategy also makes designing a variety range of hybridizations possible in comparison to the merge & fused strategies [61]. Although these strategies are utilized prevalently in neurological disorders, their principles are also applied in developing anticancer agents to achieve more efficient treatments [45, 54]. Last, a schematic overview of MTDLs deigning strategies is depicted in Fig. 1.

### Current cancer multi-target therapeutics

The current cancer treatments mostly target receptor tyrosine kinases (RTKs). They're transmembrane receptors that play a role in many cellular processes, including growth, differentiation, and metabolism [62]. RTKs are key regulators of cancer cell growth and metastasis. Dysregulation of RTK signaling can lead to a variety of human diseases, including cancer [15, 63]. Its alterations are common in a wide variety of cancers, highlighting their importance in cancer progression and making them promising therapeutic targets [64].

The JAK/STAT signaling pathway is a critical player in cancer treatment and multi-target therapy, with abnormal activation observed in various solid malignancies such as breast, lung, liver, head and neck, and stomach cancers [65]. This heightened JAK/STAT signaling has been associated with poorer prognoses, including increased recurrence rates and reduced overall survival [66]. Consequently, targeting this pathway holds promise for therapeutic interventions in cancer, showing efficacy in modulating the progression of solid tumors [67]. In summary, the JAK/STAT signaling pathway presents substantial therapeutic opportunities and is a key focus for multi-target therapy in solid malignancies.

The NF-κB pathway is another crucial regulator facilitating communication between inflammation and cancer at various levels [68]. Activation of NF-κB leads to the induction of several target genes, including those that promote cell proliferation and inhibit apoptosis [69]. Additionally, NF-κB signaling interacts with multiple other pathways, such as STAT3, AP1, interferon regulatory factors, NRF2, Notch, WNT–β-catenin, and p53. Notably, all recognized hallmarks of cancer involve NF-κB activation [70, 71]. Alterations in the NF-κB pathway are frequently observed in both solid and hematopoietic malignancies, promoting tumor cell proliferation and survival [72]. Excessive activation of the NF-κB-signaling pathway has been documented in various tumor tissues, making research on this pathway for targeted cancer therapy a significant area of interest [73]. Studies have shown that inhibition of NF-κB, either by knocking out RelA or IKK2 or by overexpressing a dominant negative form of IκBα, significantly reduces tumor volume, lowers tumor grade, and prolongs survival in mouse models [71, 74, 75].

**Fig. 2:**
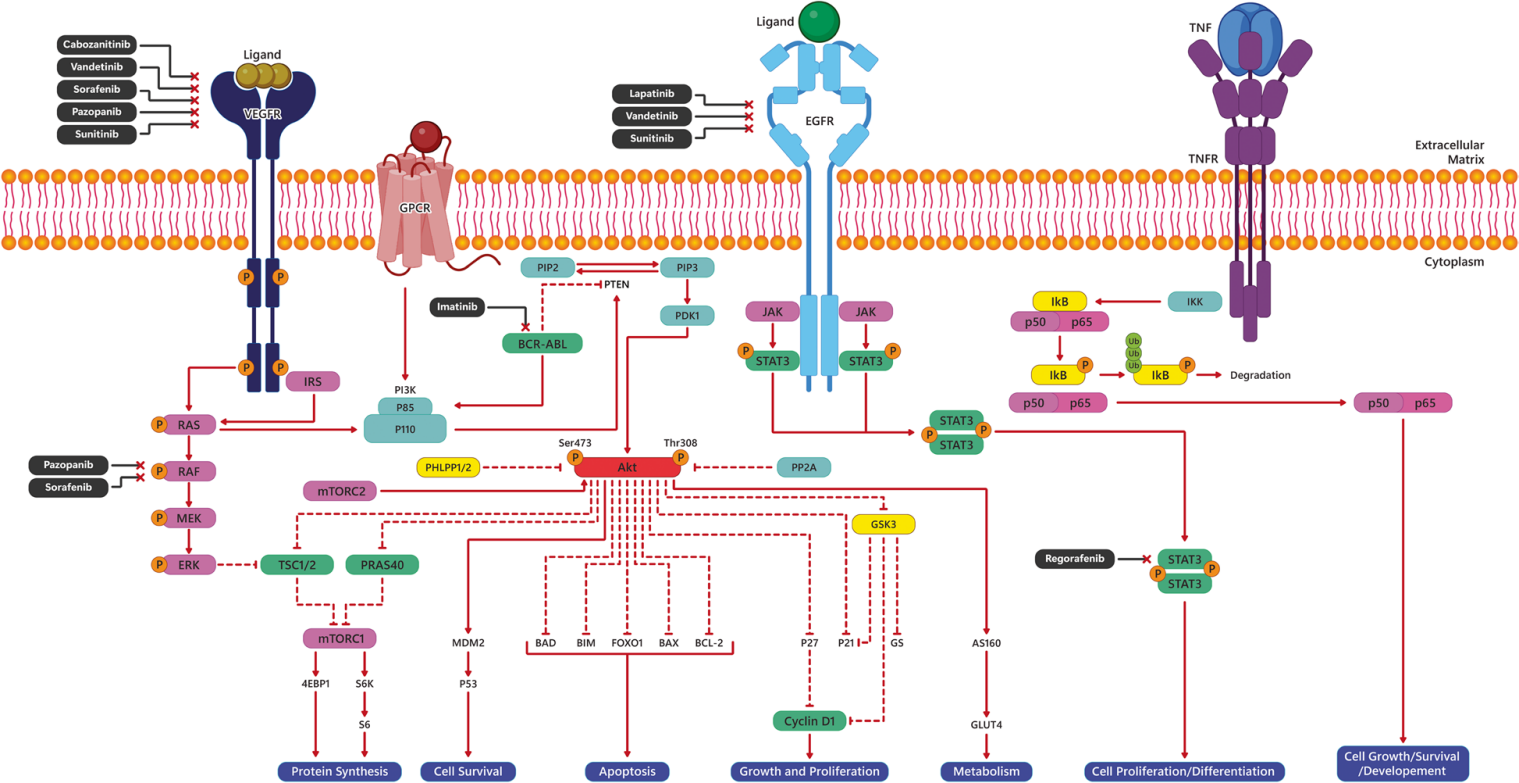
Overview of activation/inhibition of receptor tyrosine kinase (RTK)/Phosphoinositide 3-kinases (PI3K)/Akt, JAK/STAT, and nuclear factor κB (NF-κB) pathways. These pathways involve tumor cell survival, proliferation, differentiation, metabolism, apoptosis, and protein synthesis. Due to their function, these receptors and downstream molecules have been targeted over decades to increase overall survival and tumor progression inhibition. The receptors are named as an example because each signaling pathway's initiating receptors contain a variety of receptors. The black boxes describe the anticancer small molecules with their respective targets. They act through inhibiting the activity of proteins/molecules which are involved in cancer development and progression. Vascular endothelial growth factor receptor (VEGFR), G-protein coupled receptors (GPCR), epidermal growth factor receptor (EGFR), and tumor necrosis factor receptor (TNFR)

An overview of these three pathways and their involvement in cancer development, progression, and overall survival is depicted in Fig. 2. Next, we will further review multi-target drugs in cancer treatment.

### Imatinib

Imatinib (Glivec) is a first-generation multi-targeted tyrosine kinase inhibitor (TKI) that received Food and Drug Administration (FDA) approval in 2001 for malignant metastatic or unresectable gastrointestinal stromal tumors (GISTs) [76]. It's a 2-phenyl amino pyrimidine derivative that has been used to treat chronic myeloid leukemia (CML), and advanced anaplastic thyroid cancer [77, 78]. Imatinib acts by inhibiting Bcr-Abl, c-KIT, and platelet-derived growth factor (PDGF) tyrosine kinase activity through binding to their ATP-binding site [78–80]. According to the Fu et al. study [81], it indicated that imatinib's most adverse events include skin color change (55.6%) and edema (38.9%). The drug resistance related to imatinib was lysosomal sequestration that affects its target site concentration [82]. Furthermore, it has been suggested that glucose transporter (GLUT)-1 is involved in the acquisition of imatinib resistance by GIST cells, which can be overcome by combining WZB117 & imatinib [83].

### Lapatinib

Lapatinib is a first-generation quinazoline based TKI that inhibits epidermal growth factor receptor (EGFR) and human epidermal growth factor receptor 2 (HER2) reversibly [15, 84–86]. HER2 overexpression has been observed in 20 to 30% of breast cancers which is related to more aggressive disease and higher mortality [87]. Also, the overexpression of EGFR was observed in 16–36% of breast cancer cases [88]. The FDA approved lapatinib in March 2007 for treating advanced or metastatic breast cancer patients with overexpression of HER2 [89].

The lapatinib clinical trials were conducted on hormonally untreated prostate and metastatic urothelial bladder cancer, but there was no reported significant antitumor activity [90, 91]. In a phase II trial, the treatment with lapatinib did not show significant efficacy in inducing tumor regression for non-small cell lung cancer (NSCLC) in 75 patients studied [92]. On the other, lapatinib therapy is associated with a significant reduction in various forms of pain, including musculoskeletal pain, headache, bone pain, and pain in extremities, in cancer patients [93]. Its resistance is associated with a widespread reprogramming of glycolysis, which is mediated by phosphorylation and is accompanied by changes in metabolites and increased sensitivity to glycolysis inhibition [94]. The most common toxicities are diarrhea and rash, which are mostly mild to moderate in severity [95]. In most cases, symptoms are mild and do not lead to drug discontinuation [96].

### Sorafenib

Sorafenib as the first oral multi-kinase inhibitor was approved by the FDA for the treatment of patients with advanced renal cell carcinoma (RCC) in 2005, advanced unresectable hepatocellular carcinoma (HCC) in 2007, and advanced radioiodine-refractory differentiated thyroid carcinoma in 2013 [97–99]. It inhibits the activity of the serine-threonine kinases Raf-1 and B-Raf, the receptor tyrosine kinase activity of vascular endothelial growth factor receptor (VEGFR)-1/2/3, platelet-derived growth factor receptor β (PDGFR-β), c-Kit, RET, and FLT3 [15, 100, 101]. Several studies have reported diarrhea, hand-foot syndrome, rash, and fatigue as the most common adverse events related to sorafenib treatment [102–104]. Also, drug discontinuation due to intolerance or toxicities was responsible for 16% of cancerous patients [104]. Tumor cells could exhibit primary resistance or acquired resistance. In primary resistance, patients have low response rates at the initial treatment with sorafenib and gene polymorphism may play a crucial part in regulating the function of sorafenib. Many factors such as intratumor genetic heterogeneity may induce acquired resistance following sorafenib treatment, thus other treatment options should be provided [105]. HCC cell's metabolic characterization changes are also associated with their resistance to sorafenib and can be overcome by combination with aspirin [106].

### Pazopanib

Pazopanib, an oral second-generation TKI, has been approved by the FDA (2009) for RCC and soft tissue sarcoma treatment [107, 108]. Preclinical studies have suggested that pazopanib inhibits both angiogenic and oncogenic signaling pathways by VEGFR, PDGFR, fibroblast growth factor receptor (FGFR), and c-Kit inhibition [109]. It downregulates the mitogen-activated protein kinase (MAPK) signaling pathway through the inhibition of pan-RAF [110]. Interestingly, in a phase I study, 58% of patients demonstrated > 50% reduction in tumor blood flow at Day 8 of treatment, which increased to 91% at Day 22 [111]. Pazopanib is associated with several adverse effects, with hypertension as the most common one, followed by cytopenia, proteinuria, prolonged QT interval, elevated liver enzymes, diarrhea, nausea, and fatigue [103, 111, 112]. Pazopanib has indicated significant potential as a treatment option for NSCLC [113], breast cancer, urothelial carcinoma [114], thyroid cancer [115], and GIST [116].

### Sunitinib

Sunitinib, an oral multikinase inhibitor, received first approval from the FDA in January 2006 for treating advanced RCC. Subsequently, it has gained global approval for this use as well as for treating GISTs and advanced pancreatic neuroendocrine tumors in patients who are resistant or intolerant to imatinib [117]. It also has shown potential antitumor activity in various other malignancies, such as thyroid, lung, bladder, pancreatic, and esophageal carcinomas, gliomas, and sarcomas [118]. Sunitinib exerts its anti-angiogenesis effect by inhibiting RTKs including EGFR, FGFR-1, PDGFR-β, VEGFR-1/2/3, RET, FLT3, KIT, and CSF1R through competitive binding to their adenosine triphosphate (ATP) pocket [15, 119, 120]. In 2011, it was approved by the FDA for the second time to treat progressive, well-differentiated pancreatic neuroendocrine tumors [121]. There are a few ways to the drug resistance of sunitinib. One of them is autophagy-flux-associated sunitinib lysosomal sequestration which leads to the isolation of the drug from the cytoplasm in endoplasmic cells [122]. It also can promote epithelial-mesenchymal transition (EMT) in metastatic RCC cells, leading to resistance to sunitinib treatment [123]. Moreover, the most common side effects include diarrhea, nausea, asthenia, and fatigue which many studies focused on managing the drug's related drug resistance [121, 124, 125].

### Vandetanib

This second-generation TKI is another quinazoline-based orally active small molecule that exhibits potent inhibitory activity against multiple targets, including VEGFR-2 and -3, EGFR, and the rearranged during transfection (RET) receptors [126, 127]. Vandetanib significantly disrupts the EGFR-induced production of angiogenic growth factors, leading to an "indirect" impact on angiogenesis in vivo [128]. The FDA approved vandetanib in April 2011 for symptomatic or progressive medullary thyroid cancer (MTC) in patients with unresectable locally advanced or metastatic disease treatment [129]. The common adverse events are reported diarrhea, hypertension, QTc prolongation, and fatigue [130]. Among these, QTc prolongation which significantly increased during treatment with vandetanib should be well-considered due to its life-threatening effect [131]. Genetic alterations, including DNA mutations and epigenetic modifications, contribute to the resistance of medullary thyroid carcinoma to tyrosine kinase inhibition. To overcome this resistance, a potential strategy involves targeting these genetic alterations by adding further therapeutic agents [132].

### Axitinib

Axitinib is a second-generation targeted drug that selectively inhibits VEGFR 1, 2, and 3 tyrosine kinase activity [133]. It was first recommended for FDA approval by the Oncology Drug Advisory Committee (ODAC), and full approval was granted in January 2012 for the treatment of patients with advanced RCC [134, 135]. This antiangiogenic drug improved the overall survival of patients with head and neck squamous cell carcinoma [136]. The common expected side effects of this indazole-based agent are hypertension (16%), fatigue (11%), and diarrhea (11%) [137, 138]. Another point to be considered is to monitor proteinuria before initiation and periodically during treatment. So if moderate to severe proteinuria develops, the dose is reduced or even temporarily the treatment stops [134]. Generally, the majority of side effects are manageable with supportive care and dose modification [139]. So far, there has been no report of drug resistance to this drug.

### Cabozantinib

It's a second-generation multi-targeted TKI with inhibitory effects against C-mesenchymal-epithelial transition factor (C-MET), VEGFR2, RET, KIT, AXL, and FLT3, all of which play a role in the pathogenesis of liver cancer [140]. Cabozantinib was approved by the FDA for advanced RCC (2016) [141], HCC (2019) [142], and differentiated thyroid cancer (2021) [143]. It provides a substantial clinical advantage over sunitinib when used as the first-line therapy for patients with metastatic RCC [144]. Additionally, sunitinib-induced resistance can be overcome using cabozantinib in the treatment of RCC [145]. Furthermore, HCC cells overexpressed C-MET up to 40% [146], and tumor cells with low C-MET levels exhibited primary resistance to C-MET inhibitors such as cabozantinib. However, rational combinations show the potential to overcome this resistance [147]. The most reported side effects associated with the treatment were hypertension, fatigue, and diarrhea [148].

### Regorafenib

Regorafenib is a sorafenib-derived, multitargeted kinase inhibitor approved by the FDA in 2017. This second-generation TKI has demonstrated beneficial effects in the treatment of advanced HCC, metastatic colorectal cancer, and GISTs [149]. The drug targets RAS/RAF/MEK/ERK pathway by inhibiting the VEGFR, PDGFR, FGFR, KIT, and RET [150, 151]. It also can suppress AXL signaling, inhibit STAT3, and promote cell death in triple negative breast cancer [152]. Moreover, the colon cancer cell's growth and survival can be affected by regorafenib-induced generation of reactive oxygen species and synergistically enhanced oxaliplatin-induced cell growth inhibition [153]. Regorafenib's effectiveness and safety have been demonstrated in several studies. It increases patient survival and disease progression prevention which is more appealing than sorafenib due to its greater potential for RTK inhibition [154]. Also, a clinical study by Pavlakis et al. indicated regorafenib potential in the treatment of refractory advanced gastro-oesophageal cancer [155]. The side effects consist of hand-foot skin reaction, hypertension, and fatigue [156]. It has shown that HCC patients with higher topoisomerase IIα expression had shorter overall survival, but its inhibition reverses drug resistance to regorafenib [151].

### Lorlatinib

Lorlatinib is a multi-target drug and a third-generation tyrosine kinase inhibitor that can target anaplastic lymphoma kinase (ALK) and ROS1 [157]. Besides common side effects of lorlatinib including hypercholesterolemia, hypertriglyceridemia, edema, weight gain, and peripheral neuropathy [158], it has been approved twice by the FDA. The first one was in November 2018 for previously treated ALK-Positive metastatic NSCLC [159]. In March 2021, loratinib (brand name Lorbrena) was approved for the second time by the FDA for first-line treatment of patients with metastatic ALK-positive NSCLC [160]. The results of a clinical trial involving 296 patients compared the effectiveness of lorlatinib versus crizotinib. The findings indicated that lorlatinib offers advantages over crizotinib and supports its use for patients with or without baseline brain metastases [161]. Lorlatinib resistance can be caused by various mechanisms, such as ALK rearrangement in NSCLC. To overcome the resistance, some combinations such as combination with gilteritinib has been shown promising effects in silico in ALK-positive lung cancer cells [162].

### Lenvatinib

Lenvatinib is an FDA-approved (2018) TKI drug for the treatment of RCC, unresectable or advanced HCC, and radioactive iodine-refractory differentiated thyroid cancer [163]. It has been investigated due to its therapeutic effects in advanced endometrial cancer [164], adenoid cystic [165], medullary thyroid [166], and anaplastic thyroid carcinomas [167]. In a comparative clinical study, lenvatinib demonstrated similar overall survival to sorafenib in untreated advanced HCC as a first-line treatment [168]. Lenvatinib prevents tumor angiogenesis through inhibition of VEGFR-1, -2, and -3, and also blocks the proliferation of tumor cells through inhibition of FGFR-1, FGFR-2, FGFR-3, & FGFR-4, PDGFRα, RET, and c-KIT [169]. Hypertension, fatigue, weight loss, diarrhea, and nausea are the most reported adverse effects of this medication [170]. The acquired resistance with administration of lenvatinib in advanced HCC may be caused by increased activation of EGFR and insulin-like growth factor 1 receptor (IGF1R)/insulin receptor (INSR) [171]. To potentially overcome or delay resistance to the anti-tumor effects of lenvatinib, combining multiple drugs to simultaneously inhibit different angiogenic pathways could be a promising future strategy [172].

### Entrectinib

It's an orally active, small-molecule TKI for tropomyosin receptor kinases (TRK)-A/B/C, ROS1, and ALK that can cross the blood–brain barrier (BBB) [173, 174]. Entrectinib received breakthrough and priority designations from the FDA (in August 2019) and European Medicines Agency (EMA) for the treatment of neurotrophic tyrosine receptor kinase (NTRK)-positive solid tumors in adults and children with no standard options as well as adults with ROS1 + NSCLC [173]. This second-generation agent has a significant potential for treating primary and metastatic central nervous system (CNS) tumors with no adverse off-target activity [175]. The most studied adverse effects include fatigue, paresthesia, dysgeusia, myalgia, and nausea [176]. A study described a rare entrectinib resistance mechanisms in ROS1-rearranged NSCLC [177, 178]. Another study by Russo et al. [179] analyzed NTRK1 mutations that drive resistance to TRK Inhibitors. However, further assessments are also required for the occurrence percentage of the mutations.

**Fig. 3:**
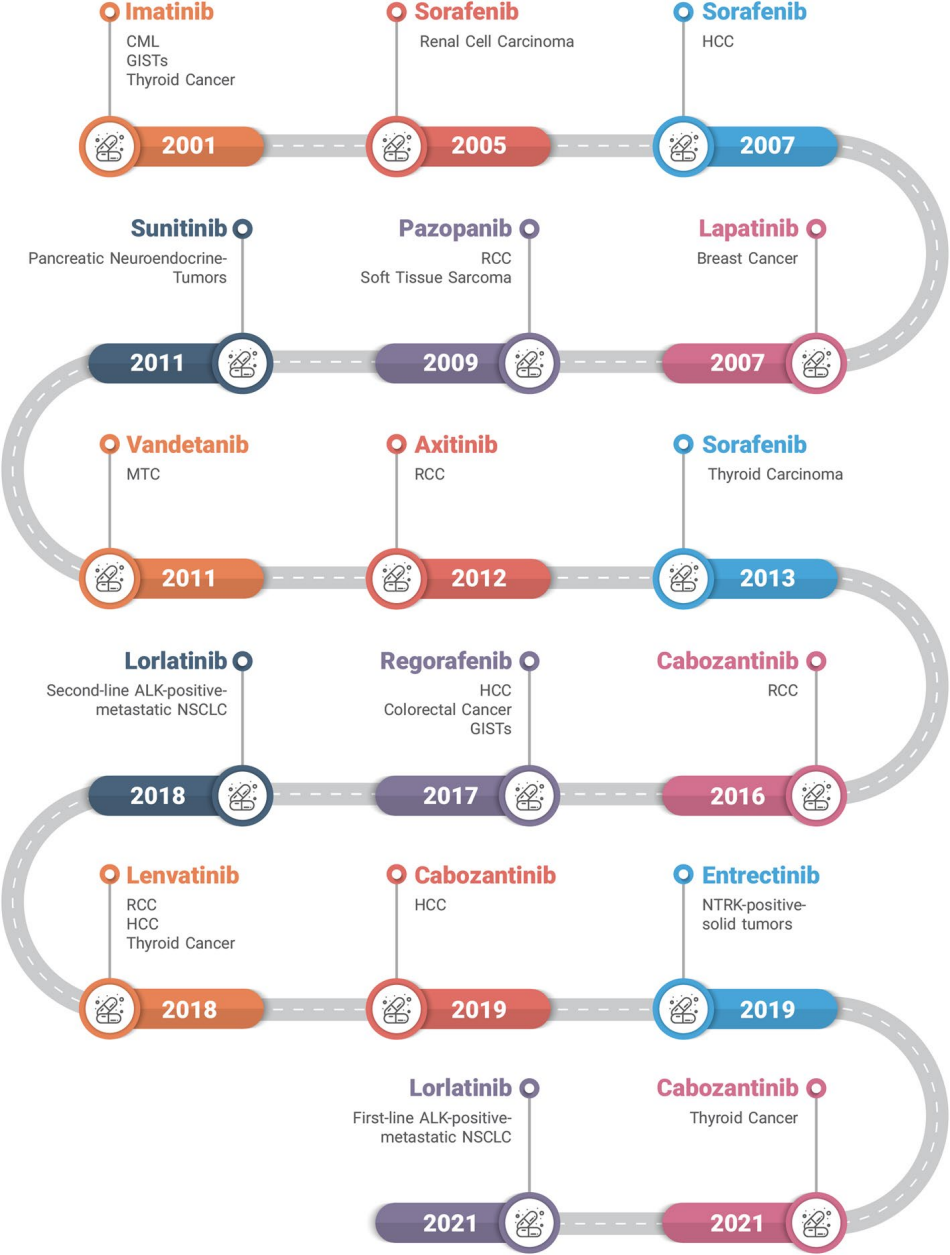
The timeline of multi-targeted drugs with their FDA approval history. Chronic myeloid leukemia (CML), renal cell carcinoma (RCC), gastrointestinal tumors (GISTs), hepatocellular carcinoma (HCC), medullary thyroid cancer (MTC), non-small cell lung cancer (NSCLC).

Last, Table 1 summed up the drugs that have been described above with their respective details. Also, the drug's FDA approval timeline has been depicted in Fig. 3. It shows that FDA-approved multi-target drugs had an upward trend which indicates their effectiveness and as a result, scientist's interests. In addition, the approval of these multi-target drugs by the FDA further underscores the potential of multi-target therapies in enhancing the outcomes of cancer treatment. Noteworthy, as it obvious most of multi-target drugs that developed in recent years are multi TKI, however, targeting novel biomarkers and different pathways at the same time using MTDLs approach would be a great opportunity to overcome RTK-induced resistance in cancerous cell [7, 180, 181].

**Table 1:** The multi-targeted drugs approved by FDA with their respective properties

No	Drugs name	Structure	FDA approval	Targets	Cancers	Adverse effects	Drug resistance	References
1	**Imatinib**		In 2001 for malignant metastatic or unresectable GISTs	Bcr-Abl, c-KIT, PDGF	CML, GIST, Advance anaplastic thyroid cancer	Skin color change, Edema	Displacement of the drug by Glut-1, Lysosomal sequestration	[75–82]
2	**Lapatinib**		In 2007 for metastatic breast cancer with overexpression of Her2	HER2, EGFR	Metastatic breast cancer	Diarrhea, rash	Glycolysis, Changes in metabolites	[15, 83–94]
3	**Sorafenib**		In 2005 for advanced RCC, In 2007 for advanced unresectable HCC, In 2013 advanced radioiodine-refractory differentiated thyroid carcinoma	Raf-1, B-Raf, c-KIT, VEGFR-1/2/3, PDGFR-β, RET, FLT3	Advanced RCC, HCC	Diarrhea, Hand-foot syndrome, Rash, Fatigue	Gene polymorphism, Intratumor genetic heterogeneity, Metabolic characterization changes	[95–104]
4	**Pazopanib**		In 2009 for RCC & soft tissue sarcoma	VEGFR, PDGFR, FGFR, MAPK, Pan-RAF	RCC, Soft tissue sarcoma, NSCLC, Breast cancer, Urothelial carcinoma, Thyroid cancer, GIST	Hypertension, Cytopenia, Proteinuria, Diarrhea, Nausea, Fatigue, QT interval	_	[103, 105–114]
5	**Sunitinib**		In 2006 for treating advanced RCC, In 2011 for progressive well-differentiated pancreatic neuroendocrine	EGFR, FGFR-1, PDGFR-B, VEGFR-2	Pancreatic neuroendocrine tumors	Diarrhea, Nausea, Fatigue, Asthenia	Autophagy-flux, Lysosomal sequestration	[115–123]
6	**Vandetanib**		In 2011 for MTC or metastatic thyroid cancer	VEGFR-2 & -3, EGFR, RET	MTC	Diarrhea, Hypertension, QTc prolongation, Fatigue	Genetic alternation, Epigenetic modifications	[124–130]
7	**Axitinib**		In 2012 for RCC	VEGFR-1, -2 and -3	RCC, head and neck squamous cell carcinoma	Hypertension, Fatigue, Diarrhea	_	[131–137]
8	**Cabozantinib**		In 2016 for RCC, In 2019 for HCC, In 2021 for thyroid cancer	c-MET, VEGFR2, RET, FLT3, KIT, AXL	Liver cancer	Hypertension, fatigue, Diarrhea	Resistance of tumor cells with low CMET levels to c-MET inhibitors	[138–146]
9	**Regorafenib**		In 2017 for advanced HCC, metastatic colorectal cancer, GISTs treatment	KIT, FGFR, PDGFR, VEGFR, RET, STAT3, AXL signaling	HCC, Metastatic Colorectal cancer, GISTs	Hand-foot skin reaction, Hypertension, Fatigue	Topoisomerase IIα high expression	[147–153]
10	**Lorlatinib**		First in 2018 for treatment of ALK positive metastatic NSCLC, In 2021 for patient with metastatic ALK positive NSCLC	ALK, ROS1	Anaplastic lymphoma kinase (ALK) and ROS1 positive NSCLC	Hypercholesterolemia, Hypertriglyceridemia, Edema, Weight gain, Peripheral neuropathy	ALK rearrangement in NSCLC	[154–158]
11	**Lenvatinib**		In 2018 for treatment of RCC, HCC and radioactive iodine-refractory differentiated thyroid cancer	VEGFR-1, -2 and -3	Endometrial cancer, Adenoid cystic, Medullary and anaplastic thyroid carcinomas, RCC, HCC	Hypertension, Fatigue, Weight loss, Diarrhea, Nausea	Increased activation of EGFR and insulin-like growth factor 1 receptor (IGF1R)/insulin receptor (INSR)	[159–167]
12	**Entrectinib**		In 2019 for treatment of NTRK positive solid tumors	TRK A,B, & C, ROS1 and ALK	Primary and metastatic CNS tumors, NTRK positive solid tumors	Fatigue, Paresthesia, Dysgeusia, Myalgia, Nausea	NTRK1 mutation with resistance to TRK inhibitor	[168–174]

Table 1 shows the FDA approval multi-target drugs in cancer treatment

### Potential targets for development of a novel multi-target cancer treatment

Colorectal, lung, and prostate cancers are among the leading causes of cancer-related deaths in the United States [182]. Therefore, targeting the potential biomarkers in these three prevalent malignancies can result in more effective multi-target agents leading to a reduction in the cancer population worldwide. Below, the potent targets (i.e. highly expressed markers or markers with the expression limited to tumor cells) have been introduced with their respective role in cancer development, progression, and survival.

### NSCLC

The NSCLC is a heterogeneous malignancy that accounts for ∼85%–87% of all lung cancers [183], which is the leading cause of cancer-related deaths worldwide [184]. The statistics recorded 1.28 million new NSCLC cases from 2010 to 2017 in United States [185].

Several proteins have been found to play crucial roles in NSCLC's development, progression, and survival. One of these markers is EGFR with an overexpression between 40–80% in advanced NSCLC patients [186]. This receptor is a member of the ErbB family that can initiate and progress the NSCLC by regulating both apoptosis and cell proliferation [184, 187]. The HER2, another ErbB family member, indicates RTK activity with an overexpression range of 2.4% to 38% [188]. Moreover, the overexpression of RTK's downstream signaling pathway molecules including phosphorylated-Akt (p-Akt) and -mTOR (p-mTOR) was observed in 78% & 46.7% of NSCLC patients, respectively [189]. The RTK/Ras/PI3K/Akt pathway promotes oncogenesis by affecting cell proliferation & growth, apoptosis, and angiogenesis, so its inhibition could be beneficial for patients [190]. In addition, the eukaryotic translation initiation factor 4E (eIF4E) is a protein with a crucial role in the initiation of protein synthesis [191]. The phospho-eIF4E expression has been found to correlate with p-Akt indicating that eIF4E activation plays a crucial role in the NSCLC progression and its upregulation has been found in 39.9% of NSCLC-diagnosed patients [189].

The AIB1 is a known potent transcriptional coactivator of estrogen receptor α that functions through direct contact with the nuclear receptor, and the overexpression (in 48.3% cases) is associated with shortened patient survival and acts as a biomarker for NSCLC patients with poor prognosis [183, 192]. The C-MET alterations are also associated with NSCLC's poor prognosis and its expression upregulates in 25–75% of diagnosed cases [193]. It is responsible for the drug resistance in most of lung cancerous cells [194]. Another tumor marker that mediates critical processes for cancer progression, such as migration, cell adhesion, and tumorigenesis is osteopontin. Its expression rate in tumor cells is 67.8%, while only 20.2% of normal lung tissues express this oncogenic protein [195, 196].

A study by Maeda et al. [197] found that carcinoembryonic antigen has the potential for targeting NSCLCs with a high level of expression (in ~ 35–60% cases) and is involved in tumor cell proliferation, adhesion, and migration [198]. The junction adhesion molecule (JAM)-A is a protein expressed on endothelial-, epithelial-, and immune cells as well as platelets [199]. The high expression of JAM-A occurred in 37% of NSCLC in comparison to the normal tissues which significantly correlates with TNM stage, lymph node metastasis, and a decrease in overall survival [200].

### Prostate cancer

Prostate cancer (PC) is the second cause of death and the first place of new cases of cancer in the United States among males [201]. The growth of prostate tumors is dependent on androgens [202] and about 80–90% of cases rely on androgens at the initial diagnosis [203]. Furthermore, the CUB domain-containing protein 1 (CDCP1) is a transmembrane protein that serves as a substrate for SRC family kinases and can cause tumor progression [204]. It's found to be overexpressed in approximately 50% of metastatic biopsies and around 30% of primary tumors [205].

The remodeling and spacing factor 1 (RSF1) protein has also been suggested to contribute to cancer progression, as its expression levels have been found to increase in more advanced pathological stages, lymph node metastasis, higher Gleason scores, and increased tumor cell proliferation [206, 207]. Detectable levels of RSF1 expression were observed in 79.2% of the 16,456 interpretable PC studied [206]. The prostate tumor overexpressed-1 (PTOV1) is a protein with 80% overexpression in patients with prostate intraepithelial neoplasia, and it's linked to prostate cancer progression. It also accumulates and alters the cancer cell's biological behavior [208]. A protein from the G-protein coupled receptors (GPCRs) family called prostate-specific GPCR 2 (PSGR2) is a receptor whose expression is restricted to human prostate tissue and exhibits distinct expressions in normal and tumor tissues [209]. The overexpression of this protein in normal and tumor tissues has a significant difference (62% of examined patients) [210].

The receptor-interacting protein kinase 2 (RIPK2) is also a predictive marker and can influence disease progression [211]. It's gained or amplified in approximately 65% of lethal metastatic castration-resistant PC and can stabilize the c-Myc transcription factor [212]. Caveolin-1 is a membrane protein highly expressed in PC and it's associated with disease progression, castration resistance, and biochemical recurrence [213]. Out of 197 cases of prostate cancer in the Chen et al. study, 111 cases were reported caveolin-1 positive (56.35%) [214]. Furthermore, elevated levels of fibroblast growth factor 8 (FGF8) in PC have been linked to reduced patient survival rates, and this association remains present even in cases of androgen-independent disease. Around 50% of clinically localized human PC express increased FGF8, while 80% or more of advanced cancers express increased FGF8 [215]. Trefoil factor 3 (TFF3) has the ability to activate ERK1/2, a crucial element of the MAPK signaling pathway. This activation ultimately leads to the promotion of tumor cell proliferation [216]. The studies have reported that TFF3 overexpression is observed in over 40% of PC cells [217, 218].

### Colorectal cancer

Colorectal cancer (CRC) is the third most frequently diagnosed and the second most fatal cancer for both males and females [219]. Globally, there is a rise in the occurrence of CRC among young adults [220]. These facts highlight the importance of new potential and novel targets for the development of anti-CRC therapeutic agents. The coiled-coil domain containing 34 (CCDC34) is a protein whose overexpression is related to CRC apoptosis reduction and metastasis enhancement and is thought to be affected via survivin, Bcl-2, N-cadherin, and E-cadherin regulation. The protein-positive rate is reported in 74.12% of patients' tissues [221]. The G-protein-coupled prostaglandin E receptor 2 (PTGER2) is a receptor that plays a crucial role in the CpG island methylator phenotype (CIMP), tumoral microsatellite instability (MSI), and survival. Out of the 516 colorectal cancers that were studied, PTGER2 overexpression was found in 169 tumors, which accounts for 33% of the total [222]. Furthermore, numerous studies have demonstrated the involvement of cyclin B1 in cancer cell differentiation, growth, apoptosis, and resistance to chemotherapy [223–226]. In 88% of the patients with CRC, cyclin B1 was found to be overexpressed compared to the non-neoplastic colorectal mucosa cells [225].

Mutations that deactivate the adenomatous polyposis coli (APC) gene and result in the increased activity of the Wnt signaling pathway play a crucial role in initiating the development of CRC and its progression [227, 228]. The APC-related mutations account for approximately 80% of CRC cases [229]. This evidence indicates the potential of targeting the Wnt signaling pathway. The overexpression of TP53 protein (TP53 +), which is involved in lymphatic and vascular invasion, is detected in 53% of stage III CRC patients [230, 231]. It also has been investigated that adjuvant chemotherapy benefit in stage III CRC is restricted to cases with low-level TP53 protein expression [231]. Moreover, the serine/arginine-rich splicing factor 3 (SRSF3) is another potential target that its high expression is associated with cell proliferation, migration, invasion, and metastasis [232]. The SRSF3 has been reported to be negative or weakly positive in 80% of patients with metastatic stage IV colorectal cancer, which was markedly related to poor survival, so it's not a good aim for advanced CRC patients [233]. But overall, the percentage of SRSF3 overexpression in CRC has been reported to be approximately 70.6% which makes it favorable especially in earlier stages [234].

**Fig. 4:**
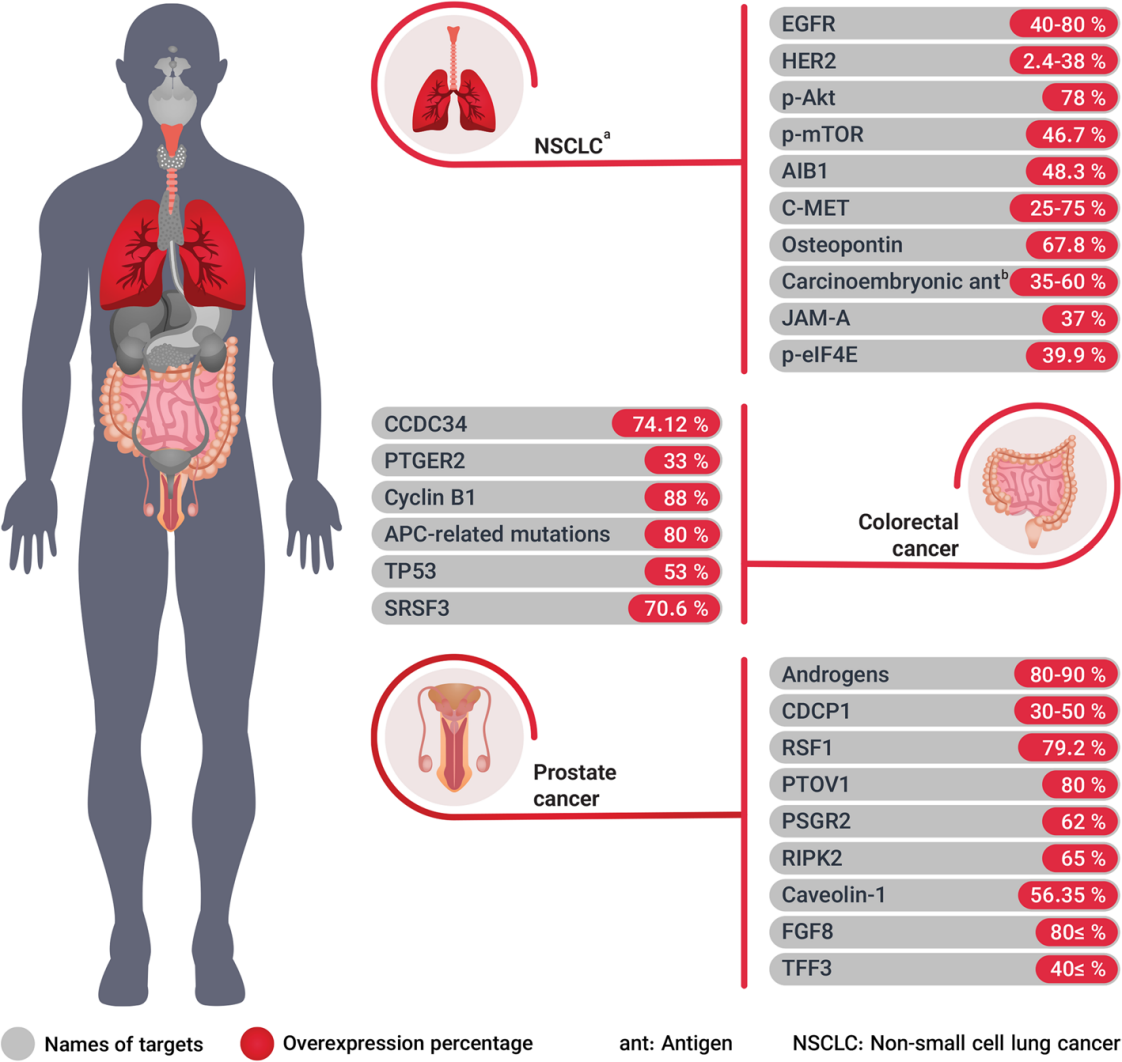
The NSCLC, colorectal, and prostate introduced selected proteins with their respective overexpression percentage as potential targets for drug development. These targets introduced due to their high overexpression which make them more desirable agents for cancer treatment

The introduced potential biomarkers for developing new anti-cancer MTDLs can be targeted whether in inhibition of the exact protein or gene downregulation. Lastly, the above mentioned malignancies with their respective biomarkers & overexpression percentages are depicted in Fig. 4.

### Future perspective

According to the 2020 statistics, there were approximately 19.3 million new cases of cancer and 10 million cancer-related deaths worldwide [1]. This indicates the emergence of exploration of the complexities and drug resistance associated with this disease. In recent decades, treatments have focused on targeting therapy which started with monotherapy and continued with combination therapies, and in recent years multi-targeted therapy has been introduced to find novel and more effective cancer treatments.

Polypharmacology involves the design and utilization of pharmaceutical agents that can act on multiple targets or disease pathways. This approach offers the potential to develop more effective drugs by specifically modulating multiple targets. Recent advancements in the computational biology approach lead to AI-based tools development for generating small molecules in silico more precisely with the employment of deep learning/reinforcement learning methods [235–237]. These web servers paved the way for the de novo design of molecules by providing knowledge-based machine learning algorithms, so drugs with more efficacy and lower toxicity become more achievable for both experts and non-experts. Then, designed small molecules can be further optimized by their affinity to selected targets using molecular docking web servers such as NeuralDock [238]. Also, for similairization of real-world interaction between ligands and their respective targets, molecular dynamics can be performed. Apart from these applications, AI-based methods provide opportunities for drug repurposing which are helpful in designing a multi-target drug [239, 240]. In addition, the identification of protein's structure and function which is necessary in the process of in silico drug designing has been facilitated by single crystal X-ray diffraction (SC-XRD), nuclear magnetic resonance (NMR), and cryo-electron microscopy (Cryo-EM) methods. On the other hand, the current multi-targeted agents are focused on small molecule deployment while the potency of peptide-based multi-targeted drugs has not been well-considered. Peptides offer significant therapeutic potential due to their high binding affinities, selectivity, specificity, and efficacy. They can also bind to surfaces, making them useful for targeting "undruggable" targets. Additionally, the diverse side chains in peptides provide a wide range of potential therapeutic targets.

All in all, MTDLs offer promising opportunities for targeting complex diseases such as cancer, either in small molecules or peptide conformations, and should be considered in hard-to-treat malignancies.

## Conclusion

Cancer treatment has become more necessary in recent years due to high rate of cancer cases worldwide. Also, treatment-induced drug resistance is another challenge that had an upward trend in recent years. These highlight the emergence of developing novel treatment strategies for combating cancer more effectively to overcome drug resistance. In the last decades, scientists moved on from monotherapy to combination therapy and recently multi-targeted agents due to the promised application provided by multi-target drugs. Moreover, the traffic of FDA-approved multi-targeted therapeutics after 2010 indicates the interest of researchers in this field. However, there are challenges in multi-target drug development such as PK/PD predictability. Recent advancements in computational biology unlocked new tools for designing hybrid compounds capable of targeting different biomarkers synergistically with desired PK features. Despite the progress in computational biology, the knowledge of drug designers is really important because the employment of these tools is not solely sufficient for achieving more effective drugs with favorable outcomes. Overall, polypharmacology, especially MTDLs, indicates reliable potential for overcoming cancer resistance.

## Data Availability

No datasets were generated or analysed during the current study.

## Abbreviations

DNA: Deoxyribonucleic acid
ER: Estrogen receptor
MTDL: Multi-target directed ligand
ADMET: Absorption, distribution, metabolism, excretion, and toxicity
PK: Pharmacokinetic
PD: Pharmacodynamics
RTK: Receptor tyrosine kinase
TKI: Tyrosine kinase inhibitor
FDA: Food and Drug Administration
GIST: Gastrointestinal stromal tumor
CML: Chronic myeloid leukemia
PDGFR: Platelet-derived growth factor
EGFR: Epidermal growth factor receptor
HER2: Human epidermal growth factor receptor 2
HCC: Hepatocellular carcinoma
RCC: Renal cell carcinoma
VEGFR: Vascular endothelial growth factor receptor
PDGFR: Platelet-derived growth factor receptor
FGFR: Fibroblast growth factor receptor
MAPK: Mitogen-activated protein kinase
NSCLC: Non-small cell lung cancer
EMT: Epithelial-mesenchymal transition
MTC: Medullary thyroid cancer
RET: Rearranged during transfection
ODAC: Oncology Drug Advisory Committee
C-MET: C-mesenchymal-epithelial transition factor
ALK: Anaplastic lymphoma kinase
IGF1R: Insulin-like growth factor 1 receptor
INSR: Insulin receptor
TRK: Tropomyosin receptor kinases
BBB: Blood-brain barrier
EMA: European Medicines Agency
NTRK: Neurotrophic tyrosine receptor kinase
p-Akt: Phosphorylated-Akt
p-mTOR: Phosphorylated-mTOR
CDCP1: CUB domain-containing protein 1
PS: Prostate cancer
RSF1: Remodeling and spacing factor 1
GPCR: G-protein coupled receptors
PSGR2: Prostate-specific G-protein coupled receptor 2
PTOV1: Prostate tumor overexpressed-1
RIPK2: Receptor-interacting protein kinase 2
FGF8: Fibroblast growth factor 8
TFF3: Trefoil factor 3
CRC: Colorectal cancer
CCDC34: Coiled-coil domain containing 34
PTGER2: G-protein-coupled prostaglandin E receptor 2
CIMP: CpG island methylator phenotype
MSI: Microsatellite instability
APC: Adenomatous polyposis coli
SRSF3: Serine/arginine-rich splicing factor 3
SC-XRD: Single crystal X-ray diffraction
NMR: Nuclear magnetic resonance
Cryo-EM: Cryo-electron microscopy
